# The Occurence of Colistin-Resistant Hypervirulent *Klebsiella* *pneumoniae* in China

**DOI:** 10.3389/fmicb.2018.02568

**Published:** 2018-10-25

**Authors:** Yang Lu, Yu Feng, Alan McNally, Zhiyong Zong

**Affiliations:** ^1^Center of Infectious Diseases, West China Hospital, Sichuan University, Chengdu, China; ^2^Division of Infectious Diseases, State Key Laboratory of Biotherapy, Chengdu, China; ^3^Center for Pathogen Research, West China Hospital, Sichuan University, Chengdu, China; ^4^Institute of Microbiology and Infection, College of Medical and Dental Science, University of Birmingham, Birmingham, United Kingdom

**Keywords:** colistin resistance, hypervirulence, *Klebsiella*, plasmid, Mcr-1 colistin resistance

## Abstract

Hypervirulent *Klebsiella pneumoniae* strains are usually susceptible to many antimicrobial agents including colistin. Here we report the isolation and characterization of several colistin-resistant hypervirulent *K. pneumoniae* clinical strains. *K. pneumoniae* strains recovered from blood samples were collected at a university hospital in China. MICs of colistin were determined using microdilution. Colistin-resistant strains were subjected to whole genome sequencing to reveal their clonal background, antimicrobial resistance determinants and virulence factors. Virulence assays were performed with strains carrying the mucoid phenotype regulator gene *rmpA* using wax moth larvae. The *pmrB* gene encoding a P344L substitution was cloned into a colistin-susceptible *K. pneumoniae* strain to examine whether the substitution confers colistin resistance. Five colistin-resistant hypervirulent *K. pneumoniae* were recovered from blood samples of patients in China, belonging to four sequence/capsular types (ST23:K1, ST412:K57, ST660:K16, and ST700:K1) and carried the virulence factor *rmpA*. Three strains had the known colistin-resistant D150G substitution in PhoQ including one ST700:K1 strain also carrying *mcr-1*. The remaining two isolates had a P344L substitution of PmrB but cloning of *pmrB* encoding the substitution into a colistin-susceptible isolate did not alter MICs of colistin, suggesting that such a substitution did not confer resistance to colistin. In conclusion, the convergence of colistin resistance and hypervirulence in *K. pneumoniae* of multiple clonal backgrounds has emerged and may warrant further surveillance.

## Introduction

*Klebsiella pneumoniae* is one of the most common bacterial pathogens isolated from clinical infections. Certain sequence and capsular types (e.g., ST23:K1) of the organism display the hypermucoviscous phenotype and carry a number of critical virulence factors such as regulators of mucoid phenotype (*rmpA, rmpA2*), aerobactin (*iucABCD, iutA*), colibactin (*clbA-R*), salmochelin (*iroN, iroBCD*), and yersiniabactin (*irp2, ybtAEPQTUX*) (Brisse et al., 2009; Holt et al., 2015). These hypervirulent *K. pneumoniae* (hvKP) are a particular threat for human health as they are able to cause severe infections in apparently healthy persons with high mortality (Shon and Russo, 2012). hvKP is commonly susceptible to many antimicrobial agents including carbapenems and colistin (Shon and Russo, 2012; Holt et al., 2015). However, some carbapenem-resistant hvKP strains have been found in China and belong to the widely distributed sequence type 11 (ST11) (Zhan et al., 2017; Gu et al., 2018) and several other sequence types, e.g., ST25 and ST65 (Yao et al., 2015). A recent study (Gu et al., 2018) has revealed that an ST11 carbapenem-resistant *K. pneumoniae* acquired a pLVPK-like virulence plasmid and therefore became hypervirulent. The combination of carbapenem resistance and hypervirulence significantly compromises options of antimicrobial agents for treating the life-threatening infections caused by these strains and therefore represents a major urgent challenge for clinical treatment, infection control and public health (Chen and Kreiswirth, 2018). Colistin is the last resort agent against carbapenem-resistant *K. pneumoniae* but colistin-resistant *K. pneumoniae* has also emerged worldwide (Olaitan et al., 2014). The combination of colistin resistance and hypervirulence will represent another major urgent challenge for human health. Here we report the isolation and characterization of five colistin-resistant hvKP clinical strains from different clonal backgrounds.

## Materials and Methods

### Strains and *in vitro* Susceptibility

Non-duplicated *Klebsiella* strains that were recovered from the blood of patients at West China Hospital, Sichuan University between December 2015 and July 2016 were collected. Initial species identification was performed using Vitek II (bioMérieux, Marcy-l’Étoile, France) and MALDI-TOF (Bruker, Billerica, MA, United States). The study has been approved by the Ethical Committee of West China Hospital with inform consent being waived. *In vitro* susceptibility tests of amikacin, ceftriaxone, ciprofloxacin, gentamicin, imipenem, piperacillin-tazobactam, sulfamethoxazole-trimethoprim, and tigecycline were performed using Vitek II. MICs of colistin were determined using the broth microdilution method of the Clinical and Laboratory Standards Institute (CLSI) (CLSI, 2017) and breakpoints of colistin defined by EUCAST^1^ were applied.

### String Tests

Colonies of the strains were stretched by an inoculation loop in the string test as described previously (Shon and Russo, 2012). The strains that formed viscous strings > 5 mm in length were considered hypermucoviscous.

### Whole Genomic Sequencing and Analysis

The strains were subjected to whole genomic sequencing using the HiSeq X10 Sequencer (Illumina, San Diego, CA, United States) with the 150-bp paired-end protocol and approximately 300 × coverage. Raw reads were processed via adapter-trimming (Illumina TruSeq DNA adapter), end-cropping (10-bp from both ends) and quality-filtering (lower than Q20) using Trimmomatic (Bolger et al., 2014). Draft genomes were then assembled using Unicycler (Wick et al., 2017) in conservative mode with other settings remaining as default, and were annotated using Prokka (Seemann, 2014). Species identification was further established by the pair-wise average nucleotide identity (ANI) between the isolates and type strains of *Klebsiella* species using the JSpecies program based on BLAST (Richter and Rossello-Mora, 2009).

Sequence types of these strains were determined using the assembled contigs to query the multi-locus sequence typing database available at http://bigsdb.pasteur.fr/klebsiella/klebsiella.html, while the capsular typing was performed for *K. pneumoniae* strains using Kaptive ^2^. Antimicrobial resistance genes were predicted using ResFinder at the Center for Genomic Epidemiology.^3^ Small insertions and deletions, as well as high quality SNPs were identified by mapping reads against the draft genome of KP925 using snippy v4.0^4^ with default settings. Core SNPs were then used as the input for phylogenetic tree inference using RAxML (Stamatakis, 2014) with a 1,000-bootstrap test. Virulence genes were identified using the database available at http://bigsdb.pasteur.fr/klebsiella/klebsiella.html. The well-studied 208,166-bp virulence plasmid pLVPK (IncHI1/IncFIB; GenBank accession no. AY378100) possesses *rmpA, rmpA2, iucABCD, iutA*, and *iroBCDN* and was selected as the reference to align contigs of the five strains in this study using BRIG (Alikhan et al., 2011).

#### Nucleotide Sequence Accession Numbers

Draft whole-genome sequences of the five strains have been deposited into GenBank under the accession no. NWCC00000000 (KP209), NWCN00000000 (KP775), NWDO00000000 (KP543), NWEN00000000 (KP925), and NWEQ00000000 (KP767).

### Cloning of *pmrB* Encoding a P344L Substitution

Two strains, KP925 and KP209, had no known chromosomal or plasmid-borne mechanisms mediating colistin resistance. However, their *pmrB* genes, which encode PmrB, part of the PmrAB two-component system, have a C1031T mutation encoding a P344L substitution. Whether the P344L substitution of PmrB could mediate colistin resistance has not been studied before. We therefore cloned the *pmrB* gene encoding the P344L substitution. The 1098*-*bp *pmrB* gene was amplified with self-designed primers XhoI_pmrAB_F (CCGCTCGAGCGGTCATTACAGCCTGATCGTGCTGGATCTCG; the restriction site is underlined) and EcoRI_pmrAB_R (CCGGAATTCCGGTCGTCCTGCTTGCCAGATAACAAACATTT) from strain KP925. The wild-type *pmrB* gene (without encoding the P344L substitution) and its promoter sequence was also amplified using the same primers from a colistin-susceptible *K. pneumoniae* strain, KP9G2, for control. Both PCR amplicons and the vector pBC SK (Stratagene, La Jolla, CA, United States) were digested using *Xho*I and *EcoR*I (NEB, Ipswich, MA, United States) and were ligated using T4 ligase (NEB) to construct pBC SK-*pmrB*^wild^ (containing *pmrB* from KP9G2) and pBC SK-*pmrB*^P344L^ (containing *pmrB* encoding the P344L substitution). pBC SK-*pmrB*^wild^ and pBC SK-*pmrB*^P344L^ were transformed into KP9G2, KP925, and KP209 by electroporation. Transformants were selected on agar plates containing 35 μg/ml chloramphenicol and *pmrB* sequences on the clonal vector in transformants were obtained using PCR with primers (M13 -20 and M13 reverse primers; Stratagene) binding to pBC SK and subsequent Sanger sequencing. To verify the expression of *pmrB*^wild^ or *pmrB*^P344L^, RNA was extracted from the transformants using the RNAprep pure cell/bacteria kit (Tiangen, Beijing, China) and cDNA was obtained using the Primescript RT reagent kit (Takara, Dalian, China), which was then used as the template for real-time PCR (RT-PCR). RT-PCR was performed using LightCycler 96 (Roche, Mannheim, Germany) with a primer (AAAAGCTGGAGCTCCACCG) binding to the pBC SK vector and another primer (GCGGCCTTTTTTCTTCTCAA) binding to *pmrB*. The 30S ribosomal subunit protein S12-encoding *rpsL* gene was used as a control for RT-PCR with the pair of primers rpsL13_F and rpsL14_F (Cannatelli et al., 2013). MICs of colistin against transformants were determined using broth microdilution of CLSI (CLSI, 2017).

### Virulence Assay

The virulence of the strains was assessed using wax moth (*Galleria mellonella*) larvae weighing 250 to 350 mg (Tianjin Huiyude Biotech Company, Tianjin, China). Overnight cultures of *K. pneumoniae* strains were washed with phosphate-buffered saline (PBS) and further adjusted with PBS to concentrations of 1 × 10^4^ CFU/ml, 1 × 10^5^ CFU/ml, 1 × 10^6^ CFU/ml, 1 × 10^7^ CFU/ml. Sixteen larvae were injected with 10 μl of inoculum using a 25-μl Hamilton syringe into hemocoel via the last left proleg (Peleg et al., 2009) and were then incubated at 37°C in plastic containers. The number of live larvae was counted every 12 h for 3 days. Two *bla*_KPC-2_-carrying carbapenem-resistant *K. pneumoniae* clinical isolates of ST11, KP10, and KP13F4, both of which had no *rmpA* and *rmpA2* genes, were used as the control. All experiments were performed in triplicate.

## Results

Among 112 *Klebsiella* strains collected from blood during the study period, five *K. pneumoniae* strains (Table 1) were found to be colistin resistant (MIC, 4 to 16 μg/ml; Supplementary Table S1) and hypervirulent (see below) and were therefore included in this study. The five *K. pneumoniae* strains were recovered from five different male patients with liver abscess, peritonitis or pancreatic cancer. There was no obvious epidemiological link among the five patients, as the hospitalization of the patients did not overlap. Three of the five strains were community acquired (as determined by days between admission and infection), all of which were associated with liver abscess, while the remaining two were hospital acquired and were not associated with liver abscess (Table 1). Three of the five strains were hypermucoviscous. Two hypermucoviscous strains belonged to ST23:K1 (Table 1), the best-known hypervirulent phylogenetic group of *K. pneumoniae* commonly associated with community-acquired pyogenic liver abscess (Brisse et al., 2009). There were 286 SNPs between the two ST23:K1 strains, suggesting that they did not share a common transmission event. Another hypermucoviscous strain was identified as ST700:K1 (Table 1). The remaining two strains formed mucoid strings < 5 mm though they were mucoid on agar plates (Table 2) and belonged to ST660:K16 and ST412:K57, respectively (Table 1). A phylogenetic tree was inferred for the five strains (Supplementary Figure S1).

**Table 1:** Characteristics of patients with bloodstream infection due to colistin-resistant hvKP.

Strain	Acquisition^1^	Patient	Age	Sex	Major diseases	Date^2^	Antimicrobial treatment	Outcome
KP767	Hospital	1	51	Male	Liver cirrhosis, primary peritonitis	2015-12	Piperacillin-tazobactam	Recovered
KP925	Community	2	68	Male	Liver abscess	2016-05	Panipenem	Recovered
KP543	Community	3	52	Male	Liver abscess	2017-03	Imipenem plus aztreonam	Recovered
KP775	Community	4	72	Male	Liver abscess	2017-04	Imipenem	Recovered
KP209	Hospital	5	59	Male	Pancreatic cancer	2017-06	Imipenem	Death

*^*1*^The strain considered as community acquired if it was recovered from a sample collected within 48 h of hospitalization. Otherwise, the strain was considered hospital acquired. ^*2*^Date refers to when the blood culture that grew colistin-resistant hvKP was collected*.

**Table 2:** Characteristics of colistin-resistant hvKP.

Strain	ST (allele no.^1^)	Capsular type	Hypermucoviscous	Colistin MIC	Colistin resistant mechanism	Antimicrobial resistance genes	Virulence factors
KP767	660 (2-1-2-1-4-1-25)	K16	-	4	PhoQ D150G	*bla*_SHV -1_, *fosA, oqxA, oqxB*	*fyuA, iroBCDN, irp2, iucABCD, iutA, kfuAB, mrkABCDFHIJ, rmpA, rmpA2, ybtAEPQTUX*
KP925	23 (2-1-1-1-9-4-12)	K1	-	8	Undefined^2^	*bla*_SHV -36_, *fosA, oqxA, oqxB*	*allABCDRS, arcC, clbBK, fdrA, fyuA, gcl, glxKR, hyi, iroBCDN, irp1, irp2, iucABCD, iutA, kfuABC, KP1, mceABCDEGHIJ, mrkABCDFHIJ, rmpA, rmpA2, ybbWY, ybtAEPQTUX, ylbEF*
KP543	412 (2-1-2-1-9-1-112)	K57	+	8	PhoQ D150G	*bla*_SHV -11_, *fosA, oqxA, oqxB*,	*iroBCD, mrkABHI, rmpA, rmpA2*
KP775	700 (10-1-17-37-12-1-9)	K1	+	16	*mcr-1* PhoQ D150G	*aph(4)-Ia, aac(3)-IVa, aph(3’)-Ia, aadA1, aadA2, bla*_SHV -1_, *bla*_CTX-M-14_, *cmlA1, dfrA1, dfrA12, fosA, fosA, floR, mcr-1, oqxA, oqxB, qnrS1, sul1, sul2, sul3, tet(A)*	*fyuA, iroBCDN, irp2, iucABCD, iutA, rmpA, ybtAEPQTUX*
KP209	23 (2-1-1-1-9-4-12)	K1	+	8	Undefined^2^	*bla*_SHV -36_, *oqxA, oqxB*	*allABCDRS, arcC, clbBK, fdrA, fyuA, gcl, glxKR, hyi, iroBCDN, irp1, irp2, iucABCD, iutA, kfuABC, KP1, mceABCDEGHIJ, mrkABCDFHIJ, rmpA, rmpA2, ybbWY, ybtAEPQTUX, ylbEF*

*^1^Allele order is gapA-infB-mdh-pgi-phoE-rpoB-tonB. ^2^The two strains did not have any known plasmid-borne colistin-resistance genes (mcr-1 to -5) and any chromosomal mutations (e.g., mutations/insertions in PhoP-Q, PmrA-B, and MgrB) that are known to confer colistin resistance*.

**FIGURE 1:**
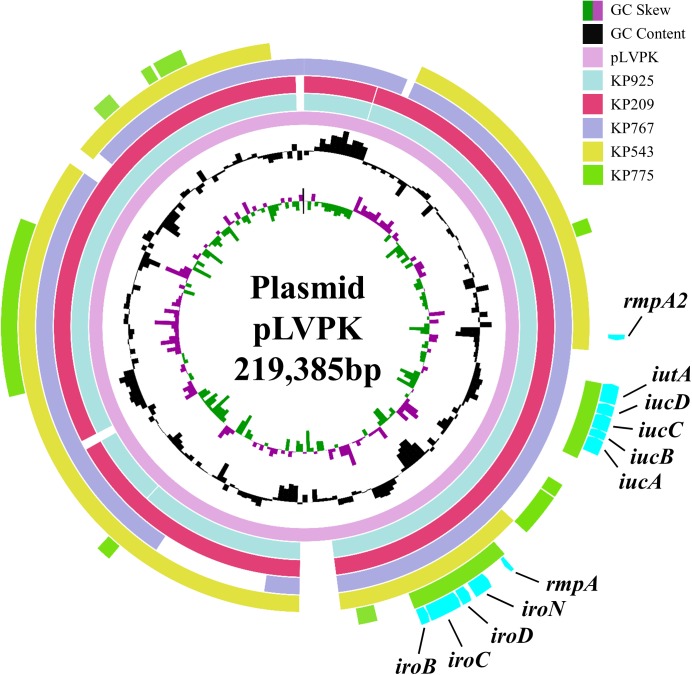
Alignment of the virulence genes-carrying plasmid pLVPK and contigs belonged to the colistin-resistant hypervirulent strains. The alignment was performed using BRIG by aligning the draft genome sequences to pLVPK (GenBank accession no. AY378100). The locations of virulence genes *rmpA, rmpA2, iucABCD, iutA*, and *iroBCDN* are indicated.

All five strains carried *rmpA* and the salmochelin-encoding *iro* cluster plus various combinations of other hypervirulence-associated genes (Table 2). Neither truncations nor stop codons were present within the open reading frames of these genes. The above virulence genes could be plasmid-borne (Brisse et al., 2009; Holt et al., 2015). Three strains aligned to almost all parts of pLVPK including the region in which the *rmpA, rmpA2, iucABCD, iutA*, and *iroBCDN* genes were located, while the remaining two strains (KP543 and KP775) aligned to most parts of pLVPK except the 16-kb region containing *iucABCD/iutA* or *rmpA2*, respectively (Figure 1).

**FIGURE 2:**
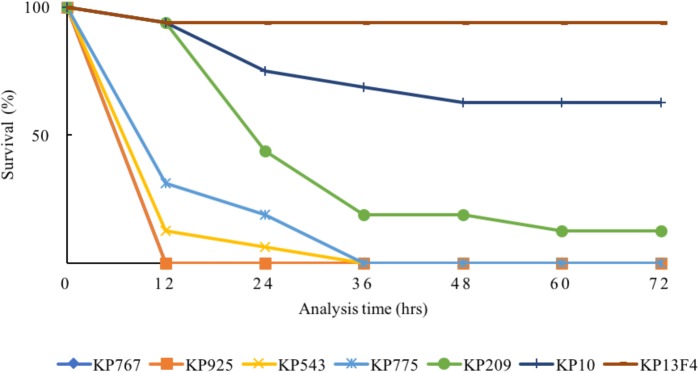
Survival of *G. mellonella* after infection by the colistin-resistant hypervirulent strains. The effect of 1 × 10^6^ CFU/ml of each hvKP isolate on survival of *G. mellonella* is shown, while those of other inoculums are shown in Supplementary Table S2. KP10 and KP13F4 were two *bla*_KPC-2_-carrying carbapenem-resistant *K. pneumoniae* clinical isolates of ST11 and were used as the control.

The 80% lethal dose at 48h of strain KP767, KP925, KP543, KP775, and KP209 against *G. mellonella* was 1 × 10^4^, 1 × 10^4^, 1 × 10^5^, 1 × 10^6^, and 1 × 10^6^ CFU/ml, respectively. Using a bacterial inoculum of 1 × 10^6^ CFU/ml, survival of *G. mellonella* at 72 h after infection was 25% with strain KP209 (hypermucoviscous, ST23:K1) and was 0% with the remaining four colistin-resistant hvKP strains including two hypermucoviscous and two non-hypermucoviscous strains, while survival was 62.5 and 93.8% with the control strains KP10 and KP13F4, respectively (Figure 2 and Supplementary Table S2). This suggests that the five colistin-resistant strains were truly hypervirulent and hypervirulence of *K. pneumoniae* is not always associated with the hypermucoviscous phenotype.

Only one (strain KP775) of the five strains carried a known plasmid-borne colistin resistance gene, *mcr-1* (Table 2). Chromosomal mutations in genes encoding PhoPQ, PmrAB, and CrrAB two-component systems and mutations or insertions in *mgrB*, which encodes a small transmembrane protein can confer resistance to colistin in *K. pneumoniae* (Poirel et al., 2017). These genes were examined in the five strains to identify mutations and insertions. Three strains had the known colistin-resistant D150G substitution in PhoQ (Poirel et al., 2017) including the one carrying *mcr-1*, while no known colistin-resistant mutations and insertions were present in the remaining two strains, KP925 and KP209. However, both KP925 and KP209 had a C1031T mutation of *pmrB*, which encodes a P344L substitution (*pmrB*^P344L^). It is unknown whether the P344L substitution of PmrB could mediate colistin resistance. Cloning of *pmrB*^P344L^ into a colistin-susceptible strain and cloning of *pmrB*^wild^ from the colistin-susceptible strain into both KP925 and KP209 were successful and *pmrB*^wild^ or *pmrB*^P344L^ was indeed expressed in the corresponding transformants. However, the introduction of *pmrB*^wild^ or *pmrB*^P344L^ did not alter MICs of colistin against the corresponding strains (Table 3).

**Table 3:** MICs colistin against of KP9G2, KP925, and KP209 and their transformants.

Strain	*pmrB* type on chromosome	*pmrB* type on pBC SK	Colistin MIC (μg/ml)
9G2	Wild		1
9G2 (pBC SK-*pmrB*^P344L^)	Wild	P344L	1
KP925	P344L		8
KP925 (pBC SK)	P344L		8
KP925 (pBC SK-*pmrB*^wild^)	P344L	Wild	8
KP925 (pBC SK-*pmrB*^P344L^)	P344L	P344L	8
KP209	P344L		8
KP209 (pBC SK)	P344L		8
KP209 (pBC SK- *pmrB*^wild^)	P344L	Wild	8
KP209 (pBC SK-*pmrB*^P344L^)	P344L	P344L	8

All five strains were susceptible to amikacin, gentamicin, ciprofloxacin, piperacillin-tazobactam, imipenem and tigecycline. Only one strain, KP775, was resistant to ceftriaxone and sulfamethoxazole-trimethoprim. Consistent with its resistance phenotype, strain KP775 had the extended-spectrum β-lactamase gene *bla*_CTX-M-14_, sulphonamide-resistance genes *sul1, sul2* and *sul3*, and trimethoprim-resistance genes *dfrA1* and *dfrA12*.

## Discussion

*Galleria mellonella* is a well-established model for testing bacterial virulence (Ramarao et al., 2012). Our virulence assays clearly suggest that the five colistin-resistant hvKP strains in this study displayed a hypervirulent phenotype. The combination of hypervirulence and carbapenem resistance has been increasingly reported recently (Arena et al., 2017; Du et al., 2018; Feng et al., 2018; Wong et al., 2018; Yao et al., 2018). However, reports on the combination of hypervirulence and colistin resistance remain scarce. Nonetheless, a hvKP strain carrying *mcr-1* has been described (Gu et al., 2016) and colistin resistance can be developed *in vitro* from colistin-susceptible hvKP strains (Choi and Ko, 2015). Recent studies have also found that in addition to the well known K1 (mainly associated with ST23) and K2 (associated with a few STs such as ST14, ST25, ST65, ST86, ST110, ST373, ST374, ST375, ST380, ST434, and ST679) (Struve et al., 2015) types, a few other types such as ST36:K62 (Feng et al., 2018), ST412:K57 (Liu et al., 2014) and ST420:K20 (Shankar et al., 2018) can also become hypervirulent. In this study, we found hvKP strains of a new type, ST660/K16, which expands the spectrum of hvKP. Among the four sequence types identified in the present study, ST23 is a common type associated with liver abscess in East Asia (Liao et al., 2014; Luo et al., 2014; Lee et al., 2016), while the remaining three STs (ST412, ST660, and ST700) remain uncommon in clinical infections. ST412 and ST660 shared five identical alleles out of the seven used for MLST and appear to belonged to the clonal group 37 (Liu et al., 2014), which contains ST37, a relatively common type associated with healthcare associated infections (Illiaquer et al., 2012; Li et al., 2017). Strains of ST412 have been found causing bloodstream infections in China (Liu et al., 2014; Ma et al., 2018) and several ST412 strains ave been recovered from throat and nasal swabs (the Klebsiella PasteurMLST database^5^). There are two ST660 strains in the database^5^, which have been recovered from sputum in China and human blood in Vietnam, respectively. ST700 is very different from ST23, ST412, and ST660 with one or two identical alleles and is also not closely related to any common types associated with clinical infections such as clonal group 258. There is only one ST700 strain (from human urine in China in the database^5^.

The five patients received a carbapenem (imipenem or panipenem) or piperacillin-tazobactam for treatment (Table 1). Four patients recovered, while one who had pancreatic cancer died. Although all strains were susceptible to piperacillin-tazobactam and carbapenems and most patients had favorable outcomes, it seems inevitable that hvKP strains with resistance to both carbapenems and colistin will soon emerge, in light of the presence of plasmid-borne colistin resistance, carbapenem resistance and hypervirulent factors.

A previous study has found that colistin-resistant mutants of ST23:K1 *K. pneumoniae* have reduced virulence compared to their colistin-susceptible progenitors with one mutation even losing the hypermucoviscous phenotype (Choi and Ko, 2015). These colistin-resistant mutants have been obtained via *in vitro* passages and selection with colistin (Choi and Ko, 2015). By contrast, the strains in the present study do not have colistin-susceptible progenitors to compare, as they were wild strains. Nonetheless, one ST23:K1 strain (KP925) in the present study was not hypermucoviscous, which is consistent with the findings of the previous study (Choi and Ko, 2015). On the other hand, both ST23:K1 still exhibited hypervirulence in the *G. mellonella* model. Therefore, we are unable to confirm the association with colistin resistance and reduced virulence in the previous study (Choi and Ko, 2015) due to the absence of the colistin-susceptible progenitors as control but such an association warrants further investigations.

Our study suggests that the P344L substitution of PmrB does not mediate colistin resistance in *K. pneumoniae*. The P344L substitution of PmrB has been seen STs other than ST23 (Pragasam et al., 2016) and not all ST23 strains have the substitution (Choi and Ko, 2015). Therefore, the colistin resistance mechanism in strains KP209 and KP925 remains undetermined. In addition to mutations or insertions in genes encoding PhoPQ, PmrAB, CrrAB, and MgrB, other mechanisms such as capsule have been suggested to be associated with colistin resistance in this species (Formosa et al., 2015). The association of capsule and colistin resistance in strains KP209 and KP925 therefore warrants further studies.

## Conclusion

In conclusion, we identified five colistin-resistant hvKP strains of different clonal backgrounds. Surveillance of colistin-resistant hvKP strains is urgently required to generate essential information for preventing their spread.

## Author Contributions

ZZ designed the study. YL and YF performed the experiments. YL, YF, AM, and ZZ analyzed and interpreted the data. ZZ wrote the manuscript. All authors have read and approved the manuscript.

## Conflict of Interest Statement

The authors declare that the research was conducted in the absence of any commercial or financial relationships that could be construed as a potential conflict of interest.

## Footnotes

1 http://www.eucast.org/

2 github.com/katholt/Kaptive

3 http://genomicepidemiology.org/

4 https://github.com/tseemann/snippy

5 http://bigsdb.pasteur.fr/
