# Supplementary material for: The Occurence of Colistin-Resistant Hypervirulent Klebsiella pneumoniae in China
Source: Front Microbiol. 2018 Oct 25;9:2568. doi: 10.3389/fmicb.2018.02568 (PMC6209640; doi:10.3389/fmicb.2018.02568)

**Supplementary file**

Table 1. MICs of antimicrobial agents against *K. pneumoniae* strains in the present study.

| Antimicrobial agent | MICs against | | | | |
| --- | --- | --- | --- | --- | --- |
|  | KP767 | KP925 | KP543 | KP775 | KP209 |
| Amikacin | ≤4 | ≤4 | ≤4 | ≤4 | ≤4 |
| Aztreonam | ≤0.25 | 0.5 | ≤0.25 | 8 | ≤0.25 |
| Ceftriaxone | ≤1 | ≤1 | ≤1 | 16 | ≤1 |
| Colistin | 4 | 8 | 8 | 16 | 8 |
| Gentamicin | ≤1 | ≤1 | ≤1 | 32 | ≤1 |
| Imipenem | ≤0.25 | 0.5 | 0.5 | ≤0.25 | ≤0.25 |
| Meropenem | ≤0.125 | ≤0.125 | ≤0.125 | ≤0.125 | ≤0.125 |
| Ciprofloxacin | ≤0.25 | ≤0.25 | ≤0.25 | ≤0.25 | 1 |
| Piperacillin-tazobactam | ≤2/4 | 4/4 | ≤2/4 | ≤2/4 | 8/4 |
| Trimethoprim-sulfamethoxazole | ≤1/19 | ≤1/19 | ≤1/19 | ≥16/304 | ≤1/19 |
| Tigecycline | ≤0.5 | ≤0.5 | ≤0.5 | ≤0.5 | 1 |

Table S2. Survival (number of larvae) of *G. mellonella* after infection by the colistin-resistant hypervirulent strains.

| Strain | Inoculum (CFU/ml) | | | |
| --- | --- | --- | --- | --- |
|  | 1 × 10^4^ | 1 × 10^5^ | 1 × 10^6^ | 1 × 10^7^ |
| KP10 |  |  |  |  |
| 12h | 16 | 16 | 15 | 9 |
| 24h | 16 | 16 | 12 | 4 |
| 36h | 16 | 16 | 11 | 3 |
| 48h | 16 | 16 | 10 | 3 |
| 60h | 16 | 16 | 10 | 2 |
| 72h | 16 | 16 | 10 | 2 |
| KP13F4 |  |  |  |  |
| 12h | 16 | 16 | 15 | 9 |
| 24h | 16 | 16 | 15 | 2 |
| 36h | 16 | 16 | 15 | 2 |
| 48h | 16 | 16 | 15 | 1 |
| 60h | 16 | 16 | 15 | 1 |
| 72h | 16 | 16 | 15 | 1 |
| KP767 |  |  |  |  |
| 12h | 0 | 0 | 0 | 0 |
| 24h | 0 | 0 | 0 | 0 |
| 36h | 0 | 0 | 0 | 0 |
| 48h | 0 | 0 | 0 | 0 |
| 60h | 0 | 0 | 0 | 0 |
| 72h | 0 | 0 | 0 | 0 |
| KP543 |  |  |  |  |
| 12h | 7 | 9 | 2 | 2 |
| 24h | 7 | 4 | 1 | 1 |
| 36h | 7 | 3 | 0 | 0 |
| 48h | 6 | 3 | 0 | 0 |
| 60h | 5 | 3 | 0 | 0 |
| 72h | 5 | 3 | 0 | 0 |
| KP775 |  |  |  |  |
| 12h | 16 | 15 | 5 | 0 |
| 24h | 13 | 15 | 3 | 0 |
| 36h | 10 | 9 | 0 | 0 |
| 48h | 9 | 8 | 0 | 0 |
| 60h | 8 | 4 | 0 | 0 |
| 72h | 8 | 4 | 0 | 0 |
| KP209 |  |  |  |  |
| 12h | 16 | 16 | 15 | 5 |
| 24h | 16 | 15 | 7 | 0 |
| 36h | 16 | 15 | 3 | 0 |
| 48h | 16 | 15 | 3 | 0 |
| 60h | 16 | 15 | 2 | 0 |
| 72h | 16 | 15 | 2 | 0 |
| KP925 |  |  |  |  |
| 12h | 8 | 6 | 0 | 0 |
| 24h | 6 | 2 | 0 | 0 |
| 36h | 3 | 0 | 0 | 0 |
| 48h | 3 | 0 | 0 | 0 |
| 60h | 3 | 0 | 0 | 0 |
| 72h | 3 | 0 | 0 | 0 |

**Figure S1. Phylogenetic tree of the five hvKP strains in this study.** The tree was inferred based on core SNPs using RAxML with a 1,000-bootstrap test. There were 286 SNPs between the two ST23:K1 strains, i.e. KP209 and KP925.


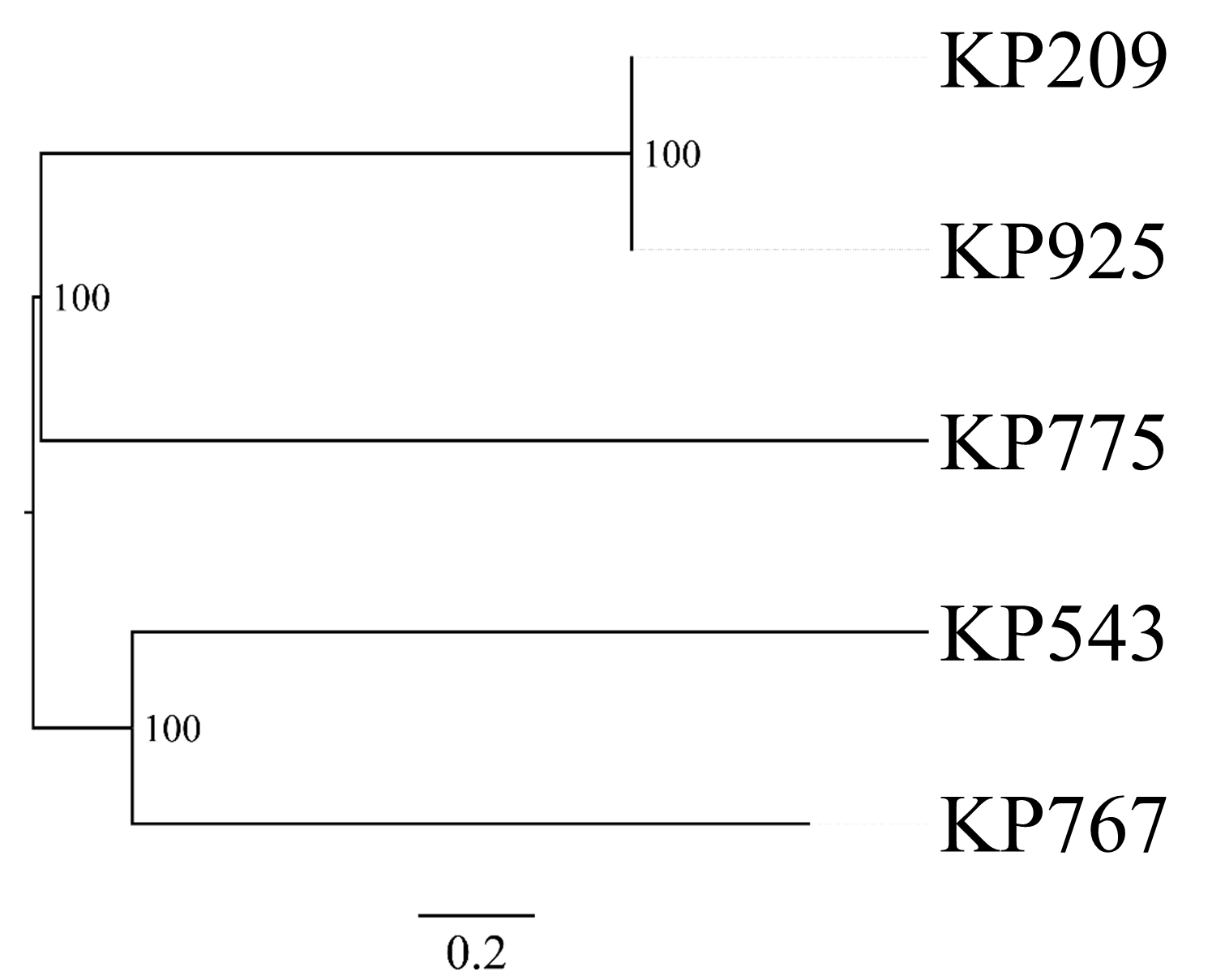

Supplement: Supplementary file 1 [file Data_Sheet_1.docx]
